# 673. Sniffing Out Adherence: Evaluation of a *Clostridioides difficile* guideline at a Tertiary Academic Medical Center

**DOI:** 10.1093/ofid/ofad500.735

**Published:** 2023-11-27

**Authors:** Tiffany Mei, Maureen Campion, Gabriela Andujar Vazquez, Kap Sum Foong, Shira Doron

**Affiliations:** Tufts Medical Center, Boston, Massachusetts; Tufts Medical Center, Boston, Massachusetts; Tufts Medical Center, Boston, Massachusetts; Tuft Medical Center, Boston, Massachusetts; Tufts Medical Center, Boston, Massachusetts

## Abstract

**Background:**

Tufts Medical Center’s (TMC) Antimicrobial Stewardship Team (AMT) in collaboration with gastroenterology (GI) developed an institutional treatment algorithm to guide appropriate inpatient management of *Clostridioides difficile* infection (CDI). The *CD* treatment algorithm was revised in April 2022 to reflect updated national clinical practice guidelines and disseminated to TMC providers. Our study aimed to assess provider compliance to TMC’s *CD* treatment algorithm.

**Methods:**

A retrospective study of adult patients hospitalized from April 15, 2022 to October 31, 2022 and tested for *CD* was conducted. We collected patient demographics, white blood cell count, serum creatinine (SC), length of stay, *CD* treatment, Infectious Diseases (ID) & GI consult, occurence of severe CDI (defined as white blood cell > 15 K/uL or SC > 1.5 mg/dL), occurrence of recurrent CDI, and immunocompromised status (defined by having had organ or bone marrow transplant, receiving immunomodulatory therapy or chronic steroids). A 2-step *Clostridioides difficile* (*CD)* testing algorithm was used. For *CD* toxin A/B & glutamate dehydrogenase antigen (GDH) indeterminate results, reflex to nucleic amplification test (NAAT) was performed only with AMT approval.

**Results:**

A total of 111 inpatients had *CD* toxin/GDH stool testing; 70 (63%) positive and 41 (37%) indeterminate. Of patients who had an indeterminate *CD* toxin/GDH, none had subsequent *CD* NAAT testing, and none receive CDI treatment. Of the others, 81 (73%), 23 (21%), and 7 (6%) were diagnosed with non-severe, severe, and fulminant CDI, respectively. The overall adherence to the institutional *CD* treatment algorithm was 58% (figure 1). More patients were appropriately treated with oral vancomycin (11, 65%) compared to those who were appropriately treated with fidaxomicin (9, 24%). Guidance adherence was (3) 38% among patients who had one or more recurrent episodes of *CD,* and none received suggested ID or GI consultation for additional CDI treatment consideration.

**Figure:**
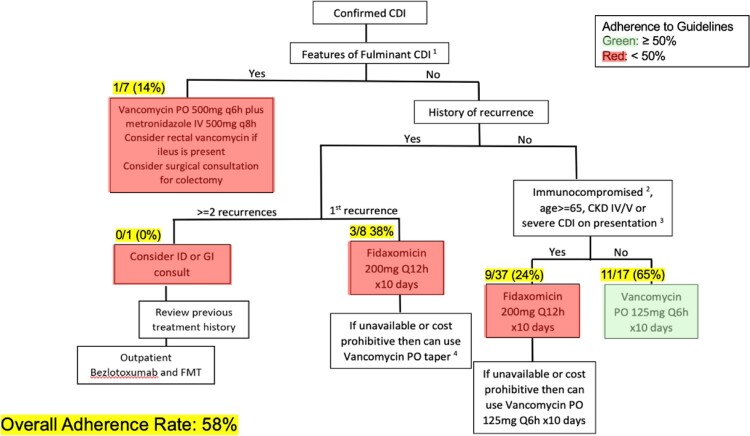

**Conclusion:**

Adherence to institutional *CD* treatment algorithm was low. This study highlights opportunities to improve prescription of first line CDI therapy, especially among patients who are at higher risk for recurrence.

**Disclosures:**

**Maureen Campion, PharmD, BCIDP**, Shinoigi: Speaker

